# *CD40* gene polymorphism and its expression in children with Kawasaki disease from North India: a preliminary case–control study and meta-analysis

**DOI:** 10.3389/fped.2023.1252024

**Published:** 2023-09-21

**Authors:** Pratap Kumar Patra, Ankur Kumar Jindal, Rashmi Rikhi, Anit Kaur, Priyanka Srivastava, Deepti Suri, Amit Rawat, Rakesh Pilania, Surjit Singh

**Affiliations:** ^1^ Department of Pediatrics, All India Institute of Medical Sciences, Patna, India; ^2^ Allergy Immunology Unit, Department of Pediatrics, Advanced Pediatrics Centre, Post Graduate Institute of Medical Education and Research (PGIMER), Chandigarh, India; ^3^ Genetics and Metabolic Unit, Advanced Pediatrics Centre, Post Graduate Institute of Medical Education and Research, Chandigarh, India

**Keywords:** Kawasaki disease, *CD40* gene, SNP *rs153045*, SNP *rs4810485*, CD40 expression

## Abstract

**Introduction:**

*CD40* gene single-nucleotide polymorphisms (SNPs) have been associated with susceptibility and development of coronary artery abnormalities (CAAs) in children with Kawasaki disease (KD) in Japanese, Chinese, and Taiwanese populations. However, data on SNPs of the *CD40* gene in patients with KD from the Indian subcontinent are not available. We studied the *CD40* gene polymorphisms and its expression in children with KD from North India.

**Methods:**

SNPs of the *CD40* gene (***rs4810485***, ***rs1535045)*** were studied using Sanger sequencing. CD40 expression was studied by flow cytometry. Meta-analysis was carried out to assess the role of both SNPs of the *CD40* gene in KD. GRADEpro GDT software (v.3.2) was used to assess the “certainty of evidence.”

**Results:**

Forty-one patients with KD and 41 age-, sex-matched febrile controls were enrolled. However, none of the alleles and genotypes of the *CD40* gene were found to be associated with KD. CD40 expression was higher in KD and in KD with CAAs compared to controls, but it failed to reach statistical significance. In a meta-analysis, the T allele of ***rs153045*** was found to be significantly associated with KD (OR = 1.28; 95% confidence interval (: 1.09–1.50; *p* = 0.002). The GRADE of evidence for this outcome, however, is of “ very low certainty.”

**Conclusion:**

The present study found no association between SNPs (***rs4810485*** and ***rs153045***) and susceptibility to KD. This could be a reflection of a modest sample size. CD40 expression was higher in KD and in KD with CAAs. In the meta-analysis, the T allele of ***rs153045*** was significantly associated with KD. Our study confirms a significant genetic heterogeneity in KD among different ethnicities.

## Introduction

Kawasaki disease (KD) is a medium-vessel vasculitis that predominantly affects young children (1, 2). The highest incidence of KD has been reported in Northeast Asian countries like Japan, South Korea, and Taiwan (3), ranging from 80 to 359 per 100,000 children below 5, and it continues to rise. Incidence data in the United States and Europe range from 15 to 20 per 100,000 children below 5. To date, no data on the nationwide incidence of KD has been gathered in India. Hospital-based studies in Chandigarh have shown that the incidence of KD is at least 5.35 per 100,000 children below 5 (4, 5). Despite more than 50 years of extensive research, the etiology of KD remains an enigma. Compared to other parts of the world, the high prevalence of KD in the Northeast Asian nations led to the speculation that the genetic endowment of an individual also plays a crucial role in KD evolution. Common genes implicated in KD are *CD40*, *ACE*, *BLK*, *CASP3*, *FCGR2A*, *FGb*, *HLA-E*, *IL1A*, *IL6*, *ITPKC*, *LTA*, *MPO*, *PD1*, *SMAD3*, *CCL17*, and *TNF* (6). However, genome-wide association studies (GWAS) found that three genes (viz., *FCG2RA*, *BLK*, and *CD40*) are consistently identified in patients with KD (7).

CD40 is a 48-kDA transmembrane protein expressed on the surface of platelets, neutrophils, monocytes, macrophages, and endothelial cells. Single-nucleotide polymorphisms (SNPs) of the *CD40* gene have been reported to predispose to KD and increase the risk of coronary artery abnormalities (CAAs) in patients with KD in Japan, China, and Taiwan (8–10). However, there are no data on *CD40* gene SNPs in patients with KD from the Indian subcontinent. Herein, we report the role of *CD40* gene SNPs, viz., ***rs153045*** and ***rs4810485***, in Indian patients with KD. We have also assessed CD40 expression on B cells in children with KD.

## Materials and methods

This case–control study was conducted at the Pediatric Rheumatology Clinic, Advanced Pediatrics Centre, Post Graduate Institute of Medical Education and Research, Chandigarh, India. The study protocol was approved by the Institute Thesis Committee and Institute Ethics Committee. The manuscript was approved by the Departmental Review Board.

The primary objective of the study was to assess the association of SNPs ***rs1535045*** and ***rs4810485*** with KD. The secondary objective was to ascertain CD40 expression on B lymphocytes in children with KD during the acute phase of the disease. We recruited 41 patients with KD, i.e., before administration of intravenous immunoglobulin (IVIg), admitted to the hospital over 1 year. We also enrolled an equal number of age-, sex-matched febrile controls from the inpatient department. Children with known chronic conditions, such as chronic kidney disease, celiac disease, nephrotic syndrome, congenital heart diseases, and neurological disorders, and children on corticosteroids or any other immunosuppressive therapy were excluded from the study.

Diagnosis of KD was based on the 2017 American Heart Association treatment guideline (11). The demographic, clinical, echocardiographic, and treatment details of patients with KD were recorded on a predesigned proforma. 2D echocardiography (2DE) was carried out by a Philips EPIQ 7 ultrasound system. Coronary arteries are considered dilated when the Z score is between >2 and <2.5. Coronary artery Z scores of >2.5 are categorized as an aneurysm. The term CAAs encompasses both dilatations and aneurysms. IVIg resistance was considered when there was persistence of or reappearance of fever at least 36 h after completion of a full single IVIg infusion (2 g/kg).

### Sample collection and DNA extraction

After obtaining written informed consent, 2 ml of peripheral venous blood was drawn by venipuncture in an ethylene diamine tetraacetate (EDTA) vial and a plain vial from each study subject and control under aseptic conditions. DNA was extracted using the DNA extraction kit and stored at −80°C until further analysis. Genomic DNA was isolated from 200 µl of EDTA blood samples using the QIAamp DNA Blood Mini Kit (Cat. No. 51106, Qiagen, Hilden, Germany).

DNA quality was determined using 1% agarose gel electrophoresis and staining with ethidium bromide. The purity of DNA was determined by measuring the optical density (OD) of samples at 260 and 280 nm using a TECAN Infinite M200 PRO with a Nanoquant plate [TECAN Group (Life Sciences and Diagnostics) AG, Switzerland], and DNA samples were stored at −80°C until further use.

### Primer designing

The reference sequence (NG_007279.1) was obtained from the National Center for Biotechnology Information Search database (NCBI). Primers were designed by Primer BLAST. Designed oligos showed highly specific PCR products.

### DNA sequencing

For genotyping of the SNPs of the *CD40* gene, DNA sequencing was done by Sanger's chain termination method. The sequencing data were obtained from an automated sequencer (ABI PRISM 3100) and analyzed using FinchTV.

### CD40 expression on B cells

The CD40 expression assay on B cells of KD patients and controls was carried out by flow cytometry. In total, 100 µl of whole blood was incubated with CD19 FITC (Beckman Coulter, Germany) alone and CD19 fluorescein isothiocyanate (FITC) with the CD40 phycoerythrin (PE) (Beckman Coulter, Germany) antibody for 15 min at room temperature in the dark. After incubation, the cells were lysed using 2 ml of NH_4_Cl solution for 15 min at room temperature in the dark. After lysis, the cells were centrifuged at 450*g* for 5 min; then, the supernatant was discarded. After the first spin, the cells were washed with 1× phosphate-buffered saline (PBS) and vortexed. After washing, cells were again centrifuged at 450*g* for 5 min. After discarding the supernatant, 300 μl of 1× PBS was added, and the cells were ready to acquire on the Navios Flow Cytometer (Beckman Coulter, Germany).

During acquisition, forward scatter (FSC) and side scatter (SSC) were adjusted to get clusters (i.e., lymphocytes, monocytes, and neutrophils) on a dot plot. Optimum CD40 expression was obtained in both tubes, i.e., CD19 alone (fluorescence minus one) and CD19 with CD40. On gating lymphocytes, CD19-positive cells labeled with PE were further gated on a separate dot plot. On gated CD19+ cells, CD40 expression was noted (labeled with FITC) on a histogram. The data were analyzed by Kaluza software.

### Statistical analysis

The data were presented as a proportion for categorical variables and mean ± standard deviation for continuous variables. Categorical variables were compared between two groups by a *χ*^2^ or Fisher's exact test. Numerical variables were compared between two groups by Student's *t*-test or the Mann–Whitney *U*-test, depending upon the type of distribution. The magnitude of effect size was expressed as risk ratio or odds ratio with a 95% confidence interval (CI). A *p*-value of <0.05 was considered significant. Microsoft Excel version 2019 was used for data entry, and R software version 3.4 was used for data analysis. For the meta-analysis, Revman 5.4 version and Meta Genyo were used. We used GRADEpro GDT software (v 3.2) to assess the certainty of evidence.

## Results

### Genotype and allele frequencies of *rs153405* in patients with KD and controls

The baseline characteristics of children with KD (29 boys:12 girls) and the control group are depicted in Table 1. The mean age of children in the study group was 4.5 ± 3.01 years, whereas it was 5.4 ± 3.02 years in the control group. The study group had 22 complete and 19 incomplete KD cases. Genotypes were in the Hardy–Weinberg equilibrium. Of 41 genotypes, the frequency of the CC genotype was the highest (22; 53.65%), followed by CT (17; 41.46%) and TT (2; 4.87%). In the control group, the frequency of the CC genotype was the highest (22; 53.65%), followed by CT (18; 43.90%) and TT (1; 2%). Frequencies of the C and T alleles were 61 (74.40%) and 21 (25.60%) in the KD group 62 (75.60%) and 20 (24.39%) in the control group, respectively. The genotype and allele frequencies of both groups are summarized in Table 2. There was no significant difference in the frequency of SNP ***rs153045*** between the study and control groups. Also, multivariate analysis revealed no genotype and allele association of ***rs153045*** with KD (Table 3).

**Table 1:** Baseline characteristics of children with KD and children in the control group.

Study variable	Kawasaki disease (*n* = 41)	Control group (*n* = 41)	*p*-value
Age	4.5 (3.02)	5.4 (3.01)	*p* = 0.3
Sex (male:female)	29:12	29:12	NA
Duration of fever in days (IQR)	10 (IQR = 5–15)	6 (IQR = 4–11)	*p* = 0.135
Coronary artery abnormalities	*N* = 5 2 = Giant coronary artery aneurysm 2 = Moderate aneurysm 1 = Small aneurysm	-	-

**Table 2:** Results of genotype analysis of *rs153045.*

Allele/Genotype	KD (*n* = 41)	Controls (*n* = 41)	Odds ratio (95% CI)	*p-*value
CC genotype	22 (56.75%)	22 (48.64%)	Ref.	0.56
CT genotype	17 (37.83%)	18 (48.64%)	1.55 (0.61–3.9)	
TT genotype	2 (5.4%)	1 (2.70%)	0.48 (0.04–5.6)	
C allele	61 (75.67%)	62 (72.97%)	0.85 (0.53–2.3)	0.77
T allele	21 (24.32%)	20 (27.02%)	0.86 (0.41–1.8)	0.70

**Table 3:** Results of bivariate and multivariate logistic regression analyses of genotypes and alleles of SNP *rs153045.*

Genotype/Allele	Odds ratio (CI) in the bivariate analysis	*p-*value	Odds ratio (CI) in the multivariate analysis	*p-*value
CC	1.34 (0.52–3.41)	0.58	1.34 (0.52–3.41)	0.54
CT	0.59 (0.25–1.55)	0.40	0.59 (0.25–1.55)	0.39
TT	1.54 (0.19–27.40)	0.68	1.54 (0.19–27.40)	0.64
C	1.46 (0.52–4.10)	0.77	1.46 (0.52–4.10)	0.75
T	0.71 (0.24–2.10)	0.53	0.71 (0.24–2.10)	0.54
Sex	1.28 (0.38–3.4)	0.62	1.28 (0.38–3.4)	0.62

### Meta-analysis of *rs153045*

We also carried out a meta-analysis of the results of our study and two previous studies (9, 10). The study reported by Onouchi et al. was not included in the meta-analysis as polymorphisms reported in this study were different from those evaluated in the present study.

**Figure 1:**
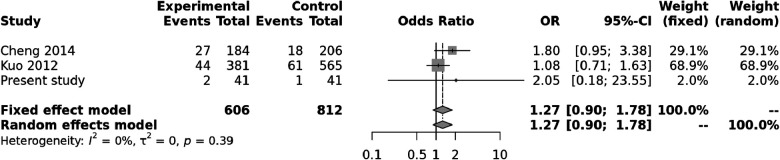
Forest plot showing the association of the ***rs153045*** genotype with Kawasaki disease (recessive model).

**Figure 2:**
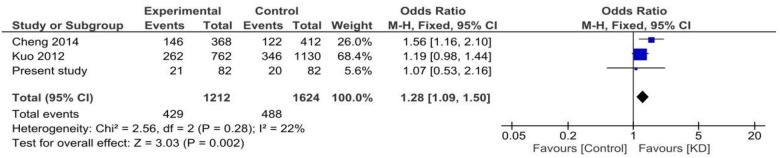
Forest plot showing the association of the T allele of ***rs153045*** with Kawasaki disease.

Both random and fixed-effect models were used. No association of genotypes of ***rs153045*** with KD was found (*p* = 0.34), and there was no heterogeneity (*I*^2 ^= 0%) (Figure 1). Similarly, no association was noted for the C allele. However, a meta-analysis found that the T allele was significantly associated with KD (*p* = 0.002) without significant heterogeneity (*I*^2 ^= 22%) (Figure 2). There was no publication bias on the funnel plot (Egger's test; *p* = 0.94). However, this evidence was of “very low certainty” (Table 4).

**Table 4:** Association of the T allele (*rs153045*) with Kawasaki disease.

Certainty assessment							Summary of findings				
Participants (studies) Follow-up	Risk of bias	Inconsistency	Indirectness	Imprecision	Publication bias	Overall certainty of evidence	Study event rate (%)		Relative effect (95% CI)	Anticipated absolute effects	
							With placebo	With the association of the T allele (***rs153045***) with Kawasaki disease		Risk with a placebo	Risk difference with the association of the T allele (***rs153045***) with Kawasaki disease
Association of the T allele (***rs153045***) with Kawasaki disease											
1,346 cases and 1,490 controls (3 observational studies)	Not serious	Not serious	Serious^a^	Not serious	None	⊕○○○ Very low	1,346 cases and 1,490 controls		OR 1.28 (1.09–1.50)	Low	
										0 per 1,000	0 fewer per 1,000 (from 0 fewer to 0 fewer)
											

OR, odds ratio.

^a^ Studied in different populations.

### Genotype and allele frequencies of *rs4810485* in patients with KD and controls

The genotype and allele frequency analyses of SNP ***rs4810485*** are depicted in Table 5. Of 41 genotypes, the GG genotype was found in 36 (87.80%) patients, with three GT (7.3%) and two TT (4.8%) genotypes in the KD group. Frequencies of G and T alleles in the KD group were 75 (91.46%) and 7 (8.54%), respectively. Likewise, the control group had 34 GG (82.93%), five GT (12.19%), and two TT (4.8%) genotypes. The frequencies of G and T alleles were 73 (89.02%) and nine (10.98%), respectively. The difference in genotype and allele frequencies between KD and control groups was not statistically significant. The results of the multivariate analysis are presented in Table 6. There was no association of genotypes and alleles of rs4810485 with KD.

**Table 5:** Results of genotype analysis of SNP *rs4810485.*

Allele/Genotype	KD (*n* = 41), *n* (%)	Controls (*n* = 41), *n* (%)	Odds ratio (95% CI)	*p-*value
GG genotype	36 (89.18%)	34 (86.48%)	Ref.	0.75
GT genotype	3 (8.1%)	5 (8.1%)	0.31 (0.03–3.17)	
TT genotype	2 (4.8%)	2 (5.4%)	1.54 (0.24–9.82)	
G allele	75 (90.54%)	73 (90.54%)	1.34 (.45–4.17)	0.57
T allele	7 (9.45%)	9 (9.45%)	1 (.31–3.2)	1

**Table 6:** Results of bivariate and multivariate logistic regression analyses of genotypes and alleles of SNP *rs4810485.*

Genotype/Allele	Odds ratio (CI) Bivariate analysis	*p-*value	Odds ratio (CI) Multivariate analysis	*p-*value
GG genotype	0.23 (0.23–4.37)	1	1 (0.22–4.43)	1
GT genotype	1.06 (0.03–3.18)	0.32	0.22 (0.01–2.97)	0.25
TT genotype	1.54 (0.24–9.82)	0.64	1.5 (0.24–10.10)	0.62
G allele	1.37 (0.45–4.17)	0.57	1.38 (0.28–6.8)	0.68
T allele	1 (0.31–3.2)	1	1.5 (0.29–3.13)	0.72
Sex	1.29 (0.48–3.44)	0.61	1.23 (0.45–3.29)	0.68

### Meta-analysis of *rs4810485*

**Figure 3:**
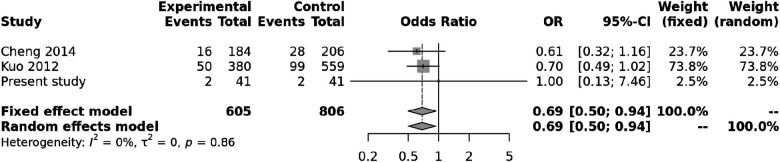
Forest plot showing the association of the ***rs4810485*** genotype with Kawasaki disease (recessive model).

**Figure 4:**
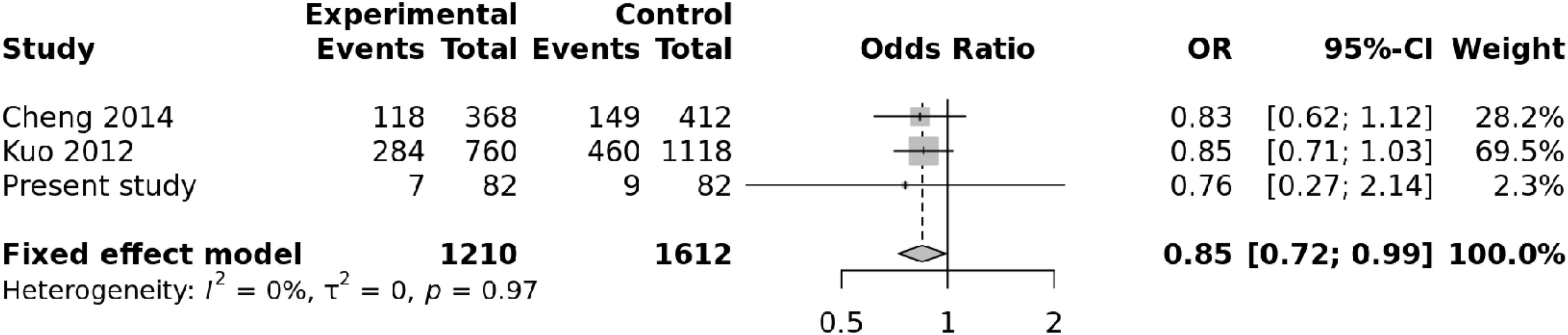
Forest plot showing the association of the T allele of ***rs4810485*** with Kawasaki disease.

We also carried out a meta-analysis of genotypes and alleles of ***rs4810485***. No association was observed for genotypes of ***rs4810485*** with KD (*p* = 0.86), and there was no heterogeneity (Figure 3). Pooled results and T alleles were also not associated with KD, *p* = 0.97, and there was no heterogeneity (Figure 4).

### CD40 expression assay

The CD40 expression assay on B (CD19+) cells was performed in patients with KD during the acute phase of the disease and in febrile controls. Flow cytometry parameters of both groups are depicted in Table 7. Although the median difference of the stained index (SI) and Δmedian fluorescence intensity (MFI) between the KD and control groups was not statistically significant, it was noted to be higher in the KD group. Similarly, compared to febrile controls, the ΔMFI was higher in patients with KD and CAAs; however, it failed to reach statistical significance (*p *= 0.21).

**Table 7:** Flow cytometry parameters of CD40.

Indices	KD group median (IQR)	Control group median (IQR)	*p-*value
Percentage expression of CD40 on B cells	99.50 (99.12–99.70)	99.59 (98.75–99.88)	0.46
Stimulation index	140.58 (37.72–417.56)	86.79 (40.95–187.79)	0.44
ΔMedian fluorescence intensity	46,779.30 (30,519.21–69,786.72)	4,24,641.68 (35,595.87–60,032.72)	0.65
ΔMedian fluorescence intensity with KD with CAAs	53,580.88 (46,402.58–56,483.33)	42,001.00 (33,100.00–59,338.00)	0.21

## Discussion

KD is the leading cause of vasculitis worldwide, and coronary artery abnormalities may develop in up to 25% of untreated patients (12). A higher incidence of KD in Japan, Korea, and Taiwan suggests that genetic components play a role in the pathogenesis of KD (13, 14). Over the last few decades, extensive research has been carried out to identify the underlying genetic component that might predispose people to KD (15).

We evaluated the CD40 expression on B cells and SNPs of the *CD40* gene (***rs1535045*** and ***rs4810485***) in a single-center cohort of patients with KD from North India. There was no significant difference in the flow cytometry expression of CD40 on B cells and SNPs in the *CD40* gene in patients with KD compared with controls. A meta-analysis of previously reported studies and the present study showed that the T allele of ***rs153045*** polymorphism in the *CD40* gene was significantly more common in patients with KD than controls.

The interaction of CD40–CD40ligand (hereafter CD40l) is believed to play a pivotal role in acute coronary syndrome (16–18). CD40–CD40l interaction leads to the proliferation of B cells, isotype switching, and release of proinflammatory cytokines (TNFα, IL10, and IL6) (19). CD40–CD0l signaling in non-hematopoietic cells also plays a crucial role in inflammation (20). However, its role in the evolution of KD (especially in KD with CAAs) is unclear.

In 2012, Onouchi et al. reported a strong association between *CD40* gene polymorphisms (***rs4813003***) and KD development (8). Subsequently, two more studies have reported the association of different SNPs of the *CD40* gene with KD (9, 10).

In the present study, the difference in genotypes of ***rs15304****5* (CC, CT, and TT) was not statistically significant between patients with KD and controls. The frequencies of C and T alleles in both groups were also not significantly different. CC was the dominant genotype, followed by CT and TT in both groups. Likewise, the C allele was the primary allele in both groups, followed by the T allele.

Genotype and allele constitution in the present study was similar to the Taiwanese population but differed from the Han Chinese population (9, 10). Cheng et al. have shown an association of ***rs153045*** with KD (10). Furthermore, the authors illustrated an association of the T allele with KD susceptibility (9). Kuo et al. have also reported a similar association of the ***rs153045*** T allele with KD. However, no association of the T allele with KD was observed in the present cohort (OR: 1.16; 95% CI: 0.54–2.4, *p* = 0.70). In the meta-analysis including these three studies, the association of the T allele with KD was significant.

A substantial difference in genotype distributions between KD patients and controls of SNP ***rs153045*** has been documented by Cheng et al. from China. Similarly, Kuo et al. have reported an association of ***rs153045*** with KD in the dominant model, although it was not significant in haplotype analysis. We observed no association of ***rs153045*** genotypes with KD in our cohort. A meta-analysis on ***rs153045*** genotypes also showed no significant association.

Regarding SNP ***rs4810485***, GG genotypes and G alleles were the most common genotypes and alleles in both groups. The genotype and allele frequencies of SNP ***rs4810485*** in our cohort differed from those in the Taiwanese and Chinese populations. The GT genotype has been reported as the most frequent genotype in both studies (9, 10). However, the G allele has been reported as the predominant allele in these studies. This finding is in accordance with our results.

In our study, the genotype and allele frequencies of ***rs4810485*** between KD patients and controls (*p* = 0.54) were not statistically different. Our results were similar to the study by Cheng et al., where the authors also reported no association between ***rs4810485*** and KD (9).

Although the risk of CAAs and KD with SNP ***rs4810485*** has been reported by Kuo et al., this association was not evident in haplotype analysis (10). Further, the meta-analysis also showed no association of any genotypes and alleles of SNP ***rs4810485*** with KD. The lack of association of genotypes of SNPs ***rs153045*** and ***rs4810485*** with KD in our cohort suggests that there is significant genetic heterogeneity in KD among different ethnicities. In addition, it is important to note that the phenotype of KD in India may be different from the rest of the world (21–23). This could be related to differences in genetic background.

To date, only two studies have explored the role of the CD40–CD40l pathway in KD (24, 25). We have previously demonstrated a significant elevation of CD40l expression in KD before the administration of intravenous immunoglobulin compared to healthy controls (24). However, it was not significantly different compared to the febrile controls. Furthermore, the soluble CD40l was not significantly different before IVIg administration. In a similar report, Wang et al. showed increased expression of CD40l on CD4 and CD8 T cells and platelets during the acute stage of KD compared to febrile controls (25). Furthermore, the CD40l expression was found to be significantly correlated with the occurrence of CAAs in KD.

To the best of our knowledge, no studies have demonstrated the CD40 expression in children with KD. We showed that CD40 expression on B cells was higher in KD and in KD with CAAs than in controls. However, this difference failed to reach a statistical significance. Our findings suggest that the interaction of CD40 and CD40l pathways may have a role in the inflammation and development of CAAs in children with KD.

The strength of our study was that genotypes were determined using Sanger sequencing, which is considered to be the gold standard. In addition, CD40 expression was also studied simultaneously. We have also carried out a meta-analysis of previously published studies to examine the relationship between different alleles and genotypes of the *CD40* gene and the risk of KD. However, the sample size of our study was admittedly modest. This is understandable because the work pertains to the DM dissertation of the first author (PP); the work had to be concluded over a definite time period. Other lacunae of our study that functional validation and haplotype analysis could not be done. Hence, extrapolation of these findings to the community may be open to questions.

In conclusion, our study confirms genetic heterogeneity in KD among different populations. *CD40* gene polymorphisms appear to be associated with (i) susceptibility to develop KD and (ii) KD with CAAs. Further studies with a larger sample size are required to validate these findings.

## Data Availability

The original contributions presented in the study are publicly available. This data can be found here: https://api.medgag.com/cases/Data-CD40-and-Kawasaki-disease/
